# Flight control and landing precision in the nocturnal bee *Megalopta* is robust to large changes in light intensity

**DOI:** 10.3389/fphys.2015.00305

**Published:** 2015-10-28

**Authors:** Emily Baird, Diana C. Fernandez, William T. Wcislo, Eric J. Warrant

**Affiliations:** ^1^Department of Biology, Lund UniversityLund, Sweden; ^2^Department of Biological Sciences, University of LethbridgeLethbridge, AB, Canada; ^3^Smithsonian Tropical Research InstitutePanama City, Republic of Panama

**Keywords:** flight control, light intensity, neural summation, *Megalopta*, *Bombus*

## Abstract

Like their diurnal relatives, *Megalopta genalis* use visual information to control flight. Unlike their diurnal relatives, however, they do this at extremely low light intensities. Although *Megalopta* has developed optical specializations to increase visual sensitivity, theoretical studies suggest that this enhanced sensitivity does not enable them to capture enough light to use visual information to reliably control flight in the rainforest at night. It has been proposed that *Megalopta* gain extra sensitivity by summing visual information over time. While enhancing the reliability of vision, this strategy would decrease the accuracy with which they can detect image motion—a crucial cue for flight control. Here, we test this temporal summation hypothesis by investigating how *Megalopta's* flight control and landing precision is affected by light intensity and compare our findings with the results of similar experiments performed on the diurnal bumblebee *Bombus terrestris*, to explore the extent to which *Megalopta's* adaptations to dim light affect their precision. We find that, unlike *Bombus*, light intensity does not affect flight and landing precision in *Megalopta*. Overall, we find little evidence that *Megalopta* uses a temporal summation strategy in dim light, while we find strong support for the use of this strategy in *Bombus*.

## Introduction

As light intensities fall, visual information becomes increasingly unreliable and nocturnal animals compensate for this by having eyes that are extremely sensitive to light (Warrant, 2008a,b). Many nocturnal insects, for example, possess superposition compound eyes, a design that greatly increases light capture compared to the apposition compound eye, which is better suited to fast vision in bright environments and is therefore more typical of diurnal insects (Land, 1981). Nonetheless, the nocturnal neotropical sweat bee *Megalopta genalis*, which relies heavily on visual information to control flight (Baird et al., 2011) and locate its nest stick (Warrant et al., 2004) in dim light, possesses apposition compound eyes. So how are these insects able to see at night? *Megalopta* elevate their photon capture by having unusually wide, light-sensitive rhabdoms, and very large facet lenses (Warrant et al., 2004; Greiner et al., 2004a). Although this increases the sensitivity of their eyes quite significantly, theoretical calculations indicate that it does not allow them to capture enough light to reliably control flight and to locate a small nest stick under the dense rainforest canopy at night (Warrant et al., 2004; Greiner et al., 2004a). Anatomical investigations suggest that *Megalopta* most likely enhances visual reliability in dim light by neurally summing visual information in the spatial domain (Greiner et al., 2004b). In addition, theoretical analyses also suggest that they may also sum this information in the temporal domain (Theobald et al., 2006), although neither possibility has been tested behaviorally.

While improving the reliability of visual information, temporal neural summation comes at the cost of decreasing sensitivity to image motion (Sponberg et al., 2015), a crucial requirement for flight control and landing in many flying insects (for a review, see: Taylor and Krapp, 2007). To maintain the precision of flight control in dim light despite a loss of temporal resolution, the insect would need to reduce the overall speed of image motion by flying slower as light levels decline. This strategy has been observed in hornets (Spiewok and Schmolz, 2006), honeybees (Menzel, 1981), and bumblebees (Reber et al., 2015). Interestingly, *Megalopta* does not seem to change ground speed in response to decreasing light intensities but instead appears to sacrifice flight performance: when returning to their nest in dim light, they fly with significantly more convoluted trajectories than when returning in brighter light, sometimes even making unsuccessful approach and landing attempts (Theobald et al., 2007). One explanation for the increased tortuosity in their flight paths is that the bees are losing temporal resolution without making any compensatory decreases in speed, something that would likely limit the precision with which *Megalopta* could control its flight and land. If *Megalopta* does indeed use temporal summation to enhance visual reliability in dim light without flying slower, we would expect flight control and landing accuracy to be significantly compromised as light levels fall. But is this the case? Here, we aim to answer this question by investigating experimentally the effect of light intensity on position control and landing in *Megalopta* and compare this with similar experiments performed on the diurnal bumblebee, *Bombus terrestris*, which most likely uses temporal summation in dim light (Reber et al., 2015).

## Materials and methods

### Animals

*M. genalis* create nests (otherwise referred to as nest sticks) by burrowing holes and tunnels into dead, broken branches, lianas and vines [typically 30–50 mm in diameter (Eickwort, 1969)], in the rainforest understory. The entrance holes to these nest tunnels are ~5 mm in diameter (Eickwort, 1969). *Megalopta* nest sticks were collected and transferred to an experimental site in the rainforest of Barro Colorado Island in Panama. The experiments were conducted in March and April 2013 (with the exception of the natural nest stick landings, which were performed in 2009, see below for details). A light meter (IL1700, International Light, USA) placed at the experimental location (2 m from the nest stick) recorded light intensity (illuminance in lux) at 1 s intervals using an electronic data logger built in-house. The time stamp of the light meter recordings was then carefully matched to the time stamp of the recordings from the camera. Trajectories of *Megalopta* returning to the nest in both experimental conditions (see below for details) were filmed under infrared illumination at 25 fps (using a Sony Handycam HDR-HC5E, Sony Corporation, Japan) during their normal foraging times, approximately 40 min both before sunrise and after sunset. The light intensities at which the flights were filmed depended on when the bees returned and were therefore not under experimental control. In some cases, more than one bee inhabited the nest stick so that two or more individual flights were recorded per session. Because we could not identify the individual bees, we therefore report the approximate number of individuals included in the data set as well as the absolute number of nest sticks.

*B. terrestris* experiments used a commercial hive (Koppert, UK) and were performed at Lund University in an indoor flight cage (2.3 m long × 2 m wide × 2 m high) during their peak activity period (between 08:00 and 14:00) at light intensities of either 19 or 190 lux. Bees returning to the nest were recorded in 30 min sessions interspersed with 30 min control periods to allow for habituation to the test condition and light intensity. For both experimental conditions (see below for details) only the first 10 flights to the nest were recorded in each session because they often occurred in quick succession—we could thus be confident that they represented 10 different individuals. Otherwise, experiments were conducted as for *Megalopta*.

For both species, different individuals were used for the two different experiments (described below).

### Statistics

Non-parametric Wilcoxon rank sum tests (z statistic) and Spearman's rank-order correlations at the 5% significance level were used for all statistical comparisons. The *r*_*s*_ statistic of the correlation test indicates the strength and sign of the relationship between -1 (perfect negative correlation), 0 (no correlation), and 1 (perfect positive correlation). Values are reported as the median and 25–75% interquartile range (iqr). Linear regression analyses were performed using the “fitlm” function in Matlab 2015a (Mathworks), which provided the F-statistic vs. constant model value and associated *P*-value.

### The effect of light intensity on flight control

**Figure 1:**
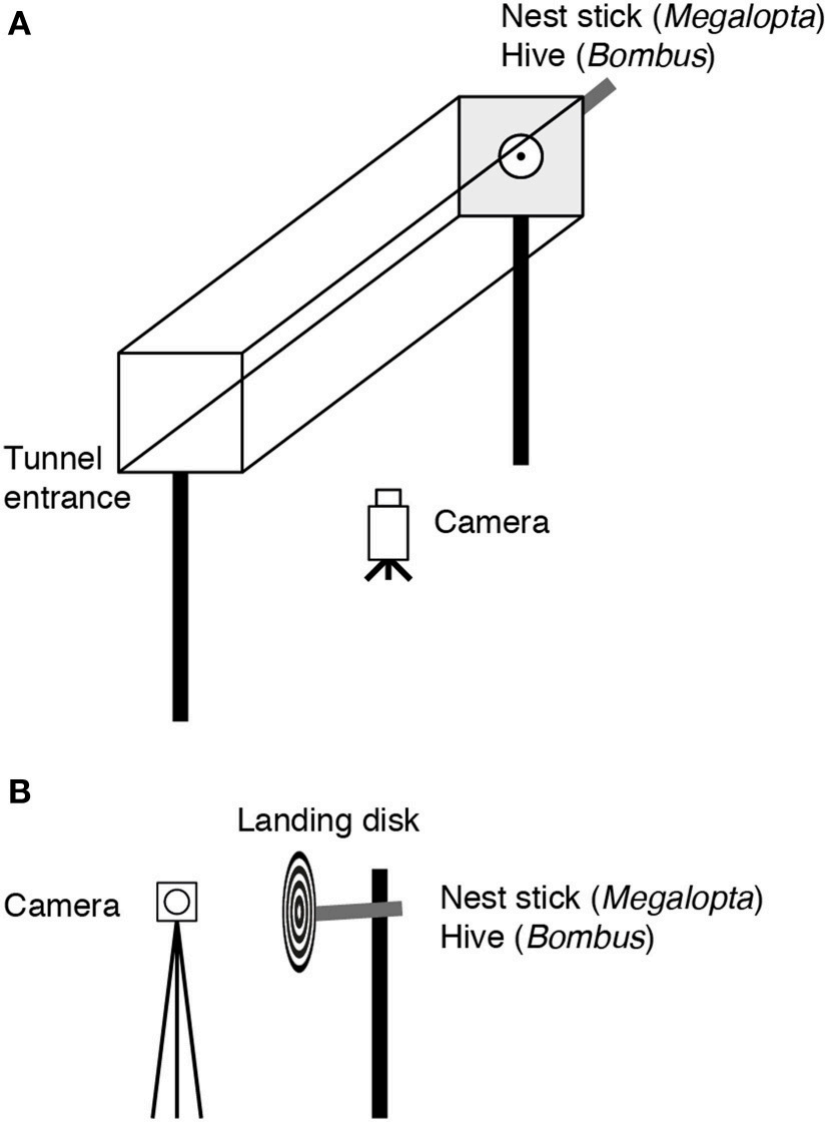
**The experimental apparatus. (A)** The experimental tunnel used to investigate the effect of light intensity on flight control in *Megalopta* and *Bombus*. Flights from the tunnel entrance to the nest were recorded using a camera mounted underneath the tunnel. **(B)** The experimental apparatus used to investigate the effect of light intensity on landing in *Megalopta* and *Bombus*. Approaches to and landings on the disk were recorded using a camera mounted to the side.

The experimental apparatus consisted of a clear acrylic tunnel, 14 cm wide × 14.5 cm high × 50 cm long, mounted 65 cm above the ground (Figure 1A). The nest was placed at an opening in one end of the tunnel at least 2 days before recording began to ensure that the bees were accustomed to flying along the tunnel to exit and enter their nest. The tunnel remained in this position for the duration of the experiment. After 2 days of habituation to the tunnel, all of the bees that flew made direct trajectories through the tunnel to the nest. The nest entrance was covered with a 5 cm diameter white disk (which had a low contrast against the sandblasted Perspex back wall) having a central 1 cm diameter hole aligned with the entrance hole of the nest. The walls of the tunnel were lined with a pattern composed of randomly distributed black and white 3 × 3 cm squares. The top panel of the tunnel was sandblasted. Flights to the nest were recorded at 25 Hz using a camera (Sony Handycam HDR-HC5E, Sony Corporation, Japan) mounted beneath the tunnel. The trajectories were analyzed over the first 25 cm of the tunnel to avoid including landing maneuvers at the nest. Fifty-one flights from 19 individuals from 11 nest sticks were recorded for *Megalopta* and 51 flights (23 flights at 19 lux and 28 flights at 190 lux) from approximately 20 individuals were recorded for *Bombus*.

Ground speed was calculated as the average of the two-dimensional distance traveled between successive frames divided by the time step between the frames (0.04 s). Accuracy of position control was calculated by finding the average lateral distance from the midline of the tunnel as well as the variance in lateral position (the iqr of lateral positions) for each flight.

### The effect of light intensity on landing

In these experiments, the flights of bees landing on either patterned disks or natural nest sticks (*Megalopta* only) were recorded (Figure 1B). Black-and-white concentric ring or radial patterns (*Megalopta* only) were printed on paper and attached to plastic disks, 10 cm in diameter with a 1 cm diameter hole at the center. The radial pattern provided strong expansion cues for bees approaching the disk while these cues were minimized in the sector pattern. In these experiments, the entrance to the nest was not placed in a tunnel but was surrounded by clear space. The disks were fitted over the nest entrance such that bees returning to their nest would have to approach and land on them. A camera mounted to the side of the disks, parallel to the trajectories of the bees, recorded the landings. Leg extension was defined as the moment when the bees began to extend their front or middle legs prior to making contact with the disk (depending on which came first). Time to contact (TC) was calculated as the time between leg extension and contact with the disk. Sixty-one landings (27 for the ring pattern, 34 for the radial pattern) from 10 individuals from four nest sticks were recorded for *Megalopta*, 58 landings from approximately 20 individuals were recorded for *Bombus*.

The natural nest stick landings for *Megalopta* were performed in March and April 2009. In these experiments, light intensity measurements (recorded in cd/m^2^) were made every 5 min using a Kodak 18% gray card reflecting incident downwelling daylight and a light meter (IL1700, International Light, USA), at a location about 2 m from the nest (as for the other *Megalopta* measurements). The nest sticks sticks were between 30 and 50 mm in diameter with a ~5 mm diameter hole that has approximately 72% contrast with the surrounding wood (Warrant et al., 2004). For the purpose of consistency and ease of comparison, these light intensity measurements were exchanged for careful intensity measurements in lux that were taken under similar conditions and at the same time of year for identical pre- and post-sunset times in later years. It is important to note that we performed statistical tests using both units and they both indicated that light intensity had no significant effect on landing precision in this experiment.

## Results

### Changes in light intensity affect flight control in *Bombus* but not in *Megalopta*

**Figure 2:**
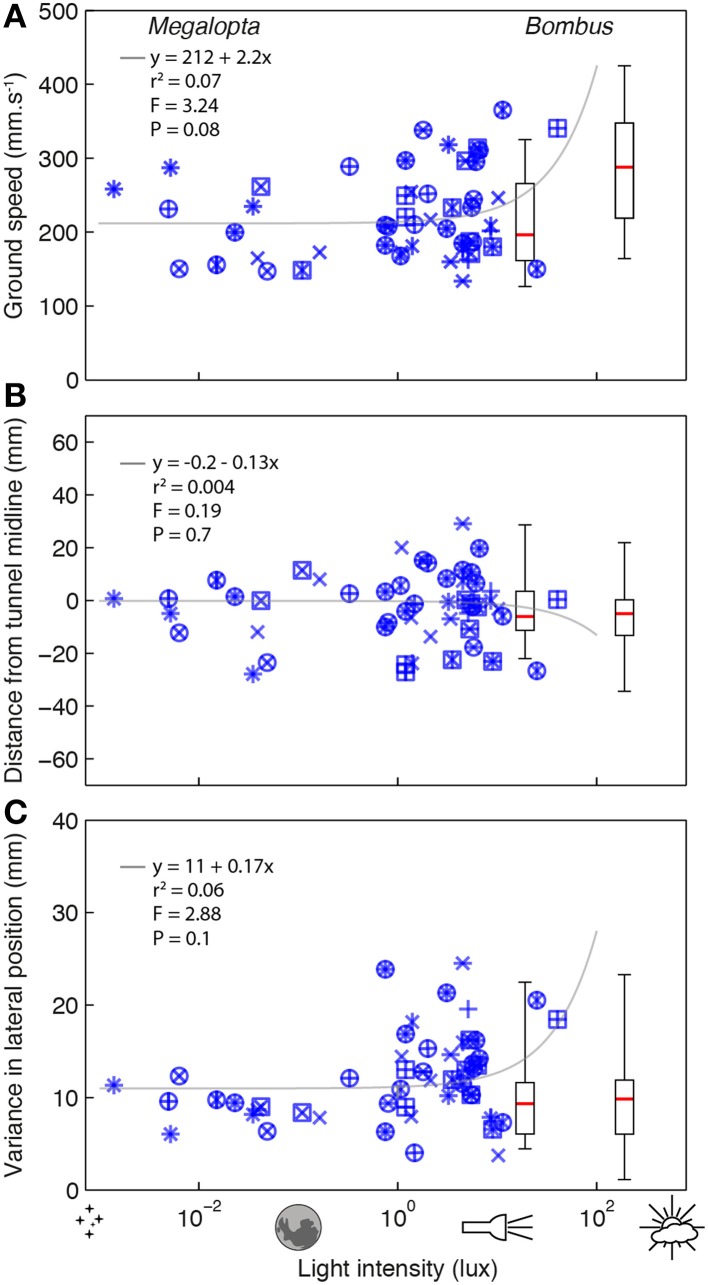
**The effect of light intensity on flight control in *Megalopta* and *Bombus.*** The effect of light intensity on ground speed **(A)**, median lateral position **(B)**, and variance (inter-quartile range) in lateral position **(C)** of *Megalopta* (blue stars; 51 flights, 19 individuals, 15 nests—different symbols indicate data from different nests) and *Bombus* [box plots; 23 flights (19 lux), 28 flights (190 lux), ~20 individuals] flying along an experimental tunnel (140 mm wide) at different light intensities. The boxes indicate the 25–75% quartile of the data, the red line indicates the median and the whiskers show the extent of the data. Gray lines indicate a linear regression analysis of the *Megalopta* data; details of the analysis and the statistical comparison (*F*-value) against a constant model are provided in each plot.

To investigate the effect of light intensity on flight control in *Megalopta* and *Bombus*, we recorded the trajectories of bees flying through an experimental tunnel at different light intensities. Flights of *Megalopta* were recorded over a range of light intensities between 0.0014 and 40.4 lux (this could not be experimentally controlled as it was determined by when the bees chose to return to their nest after a foraging trip). Despite the four log units of difference, light intensity did not have a strong effect on ground speed (*r*_*s*_ = 0.24, *P* = 0.09, *n* = 51; Figure 2A) nor the absolute lateral position (*r*_*s*_ = −0.06, *P* = 0.69; Figure 2B). There is a suggestion, however, that the within-flight variance of lateral position is weakly affected by light intensity (*r*_*s*_ = 0.26, *p* = 0.06; Figure 2C). This result is also reflected in the linear regression analyses of the data and the statistical comparison with a constant relationship between the variables (details of these analyses are provided in each subplot of Figure 2). In contrast to *Megalopta*, the ground speed of *Bombus* flying at higher light intensities of 190 or 19 lux (the bumblebees were reluctant to fly in lower light intensities) was significantly affected by light intensity (*r*_*s*_ = 0.46, *P* < 0.001, *n* = 50; Figure 2A), although the absolute lateral position (*r*_*s*_ = 0.07, *P* = 0.63; Figure 2B) and the variance in lateral position was not (*r*_*s*_ = 0.05, *p* = 0.76; Figure 2C).

### Changes in light intensity affect landing control in *Bombus* but not in *Megalopta*

**Figure 3:**
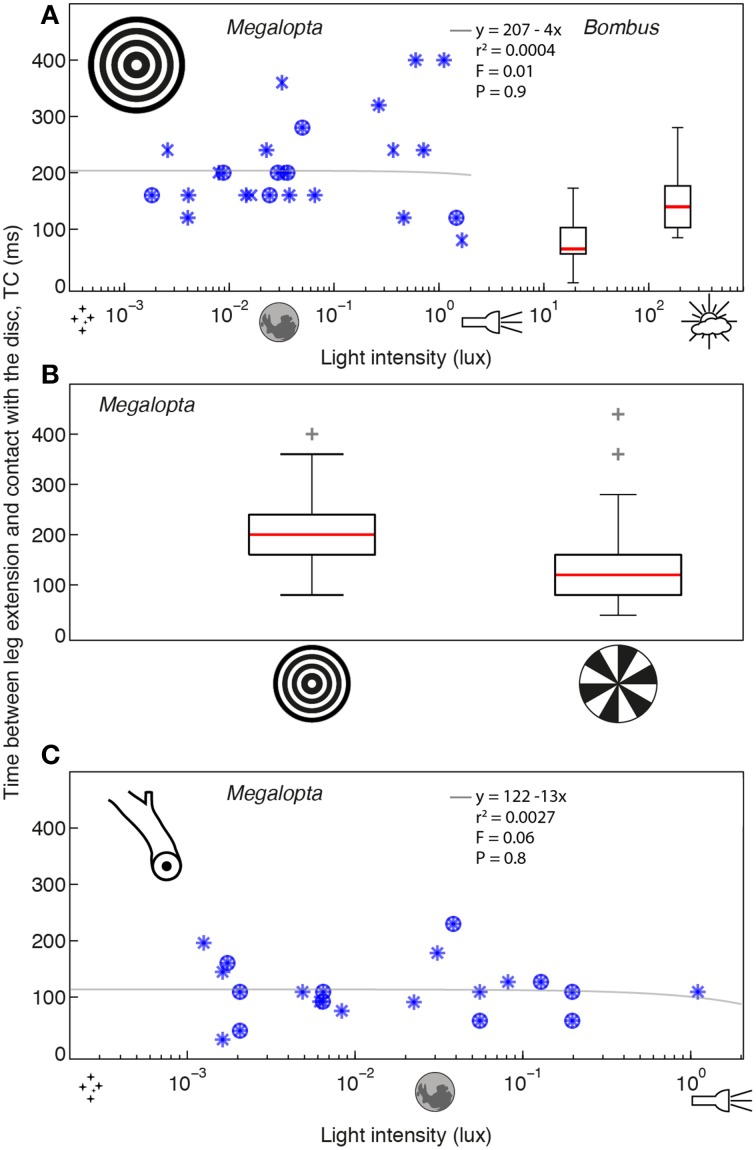
**The effect of light intensity on the timing of leg extension when landing in *Megalopta* and *Bombus*. (A)** The effect of light intensity on the time between leg extension and contact (TC) with a concentric ring pattern (inset) in *Megalopta* (blue stars, 27 landings, 10 individuals, 4 nests—different symbols indicate data from different nests) and *Bombus* [box plots, details as in Figure 1; 21 landings (19 lux), 37 landings (190 lux), ~20 individuals]. **(B)** The effect of visual expansion cues on TC in *Megalopta* [ring pattern (inset): 27 landings, radial pattern (inset): 34 landings]. Box plot details as in Figure 1 indicate the distance between the lower and upper quartile values, red lines indicate the median, whiskers indicate the entire spread of the data and red crosses indicate outliers. **(C)** The effect of light intensity on TC for a natural nest stick (inset) in *Megalopta* (23 landings, 4 individuals, 2 nests—different symbols indicate data from different nests). Gray lines indicate a linear regression analysis of the *Megalopta* data; details of the analysis and the statistical comparison (*F*-value) against a constant model are provided in each plot.

To investigate the effect of light intensity on the precision of landing (measured in terms of the timing of the leg extension response), we recorded the final stage of return flights to the nest in *Megalopta* and *Bombus* under different light intensities. *Megalopta* landings on a concentric ring pattern (which provides strong visual expansion cues) were recorded over a range of light intensities between 0.0018 and 3.58 lux (once again, this was not under experimental control but rather determined by when the bees returned to their nest after a foraging trip). Over this range, light intensity did not affect the time between leg extension and contact with the surface, TC (*r*_*s*_ = −0.02, *P* = 0.91, *n* = 27; see also details of the linear regression analysis in Figure 3A). In contrast, a single order of magnitude change in light intensity from 190 to 19 lux was sufficient to significantly affect TC in *Bombus* landing on the same pattern (*r*_*s*_ = 0.4, *p* = 0.0016, *n* = 58; Figure 3A). Because only light intensity varied in this experiment, this result suggests that visual information is important for initiating the leg extension response in *Bombus* but that it may not play such an important role in *Megalopta*.

To examine if visual cues are used to regulate the timing of the leg extension response in *Megalopta*, we compared TC for a concentric ring pattern, which provides strong visual expansion cues, with TC for a radial pattern, which provides only weak expansion cues over a similar range of light intensities (0.00094–19.94 lux). If the bees use visual expansion cues to initiate a leg extension, we expect that it will be initiated later (that is, TC will be reduced) for the radial pattern because the bees will receive little information about the distance to the surface. A TC lower than that obtained for the ring pattern (which we assume to represent optimal timing for landing) would indicate that landing has become less precise. Our results showed that TC was affected by the visual pattern (Wilcoxon rank sum, *z* = 3.3, *P* < 0.0001, *n* = 61; see also details of the linear regression analysis in Figure 3B), with leg extension occurring earlier for the ring compared to the radial pattern (ring: 200 [80] ms; radial: 120 [80] ms, median [iqr]). This decrease in TC for the radial pattern indicates that *Megalopta* becomes less precise when visual expansion cues are removed, suggesting that these cues are important for coordinating the timing of leg extension during landing.

To investigate if TC in *Megalopta* is robust to light intensity under more natural conditions, we filmed landings on unmodified nest over a range of light intensities between 0.0007 and 0.95 lux. Again, we found no effect of light intensity on TC (*r*_*s*_ = −0.05, *p* = 0.81, Figure 3C), although the average of 116 [46] ms is lower than the value of 200 [80] ms recorded for the much larger ring pattern, suggesting that the size and visual saliency of the landing surface is another factor that affects the timing of leg extension in *Megalopta*.

## Discussion

In this study, we investigate the effect of light intensity on flight control and landing in a nocturnal (*Megalopta*) and diurnal (*Bombus*) bee species. Overall, we find that flight control and landing precision in *Megalopta* is not strongly affected by light intensity, even over a five orders of magnitude decrease from twilight down to illumination levels approaching starlight. In contrast, ground speed and landing precision in *Bombus* decrease significantly over just a single order of magnitude decrease in light intensity, from illumination levels similar to an overcast day to those experienced just before twilight.

The finding that light intensity does not have a strong effect on ground speed in *Megalopta* is consistent with previous findings (Theobald et al., 2007), despite the large methodological differences between analysing natural return flights in the earlier study and analysing flights in an experimental tunnel in the present study. Does this lack of dependence of ground speed on light intensity come at the cost of flight performance in *Megalopta*? Surprisingly, we found that flight accuracy, at least in terms of positioning between the walls of a tunnel, does not worsen even over a five orders of magnitude decrease in light intensity. Despite the increased sensitivity afforded by their optical specializations (Greiner et al., 2004a), the ability to maintain the same level of flight control precision over such a broad range of intensities strongly suggests that *Megalopta* rely on neural summation strategies to improve visual reliability in dim light. The lack of a change in ground speed in combination with a negligible effect on precision makes it unlikely that *Megalopta* rely heavily on temporal neural summation strategies to control flight in dim light but that they more likely rely heavily on spatial summation strategies to do this.

In contrast to *Megalopta*, we observe a strong effect of light intensity on flight control in *Bombus*, even over a single log unit change in light intensity. These findings are consistent with previous work (Reber et al., 2015) and suggest that, as light intensities fall, *Bombus* use neural temporal summation to improve visual reliability and that they compensate for the subsequent loss of temporal resolution by flying more slowly, as hornets (Spiewok and Schmolz, 2006) and honeybees (Menzel, 1981) also appear to do. This compensatory decrease in ground speed allows them to obtain enough visual information to continue to control their position accurately.

To date, all investigations into the effect of light intensity on flight control in insects have focussed primarily on how light intensity affects ground speed. However, insects must control more than their speed to be able to fly safely in dim light. One of the most challenging behaviors that flying insects must perform is landing. To orchestrate a safe and efficient landing, flying insects need to determine the moment when they will contact the surface so that they can extend their legs in time. One cue that stimulates this leg extension response in tethered flies is the apparent rate of image expansion generated by the surface, which is used to measure the relative distance to the surface and the TC (Goodman, 1960; Wehrhahn et al., 1981; Borst, 1986; Borst and Bahde, 1986)—once this apparent rate of expansion reaches a certain threshold value, the leg extension reflex is initiated. To investigate if changes in light intensity affect landing precision in *Megalopta* and *Bombus*, we analyzed the effect of light intensity on the timing of the leg extension reflex.

As with the other parameters of flight control discussed above, the timing of the leg extension reflex is not affected by light intensity in *Megalopta*, while in *Bombus* precision is clearly lost and they extend their legs much later (i.e., closer to the nest) when light intensity decreases. One possible explanation for the lack of observeable effect of light intensity on TC in *Megalopta* is that the leg extension response is not mediated by visual cues. However, when we tested the effect of removing expansion cues from the landing surface, we find that *Megalopta* extend their legs later, suggesting that visual cues do indeed play an important role in the control of landing and that the neural summation mechanisms they employ do not affect their ability to measure the rate of expansion of optic flow cues generated by the landing surface, despite a four orders of magnitude decrease in light intensity (note that the bees in the tunnel experiments flew over five orders of magnitude difference in light intensity while in the landing experiments the flights were distributed over four orders of magnitude).

Although our results show that the timing of the leg extension response in *Megalopta* is not affected by light intensity, the patterns that we used in the experiment were not representative of the natural landing surface of an unmodified nest stick, for which the only strong contrast cues are provided by the edge of the stick and the dark entrance. Although the timing of the leg extension was reduced for landings at the nest stick in comparison to landings on the disk, suggesting that the size and visual saliency of the landing surface might be an important cue, we find no effect of light intensity in this situation either. This result further supports our finding that flight control and landing precision in *Megalopta* is extraordinarily robust to large changes in light intensity.

Considered together, the results of this study reveal that the visual control of flight and landing in *Megalopta* is not affected by large changes in light intensity, even at intensities similar to a moonless clear night sky (~10^−3^ lux, according to our own measurements). Under similar experimental conditions (but under much brighter limiting light levels), *Bombus* fly more slowly and the time between leg extension and landing decreases—elevating their risk of colliding with the landing surface (an event that was frequently observed at low light levels)—even for a decline in light intensity of just a single order of magnitude. These findings suggest that the neural summation strategies employed by these two species are fundamentally different. The reduction in ground speed and landing precision observed in *Bombus* as light levels fall strongly supports the hypothesis that they rely on neural temporal summation mechanisms to obtain enough visual information to see in dim light. In contrast, *Megalopta* do not fly more slowly and nor does their flight accuracy appear to suffer, even over a very large range of light intensities. This strongly implies that their temporal resolution does not vary with light intensity and that spatial summation is instead employed to ensure sufficient visual reliability to control flight at night. At first glance, these findings appear to contradict those of Theobald et al. (2007), who showed that flight trajectories become more tortuous as light intensity decreases, suggesting a loss of precision. Our results suggest, however, that the apparent loss of accuracy is not due to a decrease in the accuracy of flight control *per se* but rather to a decrease in the ability of *Megalopta* to accurately locate the nest stick due to increased spatial summation (as evidenced by the shorter time between leg extension and landing at a natural nest, Figure 2C). Nonetheless, these bees could use coarser spatial landmarks in the rainforest to systematically home in on the general vicinity of their nest stick, thus eventually allowing them to locate it.

Here, we show that the neural summation strategies of *Megalopta* are adequate for the fine control of flight and landing while also enabling them to navigate over large distances back to their nest across a broad range of light intensities, which change rapidly and somewhat unpredictably in their equatorial habitat (Endler, 1993). An improved understanding of how *Megalopta* increase their visual sensitivity without sacrificing flight precision will not only be important for understanding how animals use neural adaptations to optimize sensory information when signal-to-noise ratios are low but also for the development of artificial visual guidance systems that are effective in dim light.

## Funding

Air Force Office of Scientific Research/European Office for Aerospace Research and Development (grant numbers FA8655-07-C-4011, FA8655-08-C-4004). The Swedish Foundation for Strategic Research (2014-4762).

### Conflict of interest statement

The authors declare that the research was conducted in the absence of any commercial or financial relationships that could be construed as a potential conflict of interest.
